# Recurrent *DNMT3B* rearrangements are associated with unfavorable outcome in dicentric (9;20)-positive pediatric BCP-ALL

**DOI:** 10.1038/s41375-023-02058-w

**Published:** 2023-10-16

**Authors:** Željko Antić, Alena van Bömmel, Konstantin Riege, Jana Lentes, Charlotte Schröder, Julia Alten, Cornelia Eckert, Lara Fuhrmann, Doris Steinemann, Lennart Lenk, Denis M. Schewe, Martin Zimmermann, Martin Schrappe, Brigitte Schlegelberger, Gunnar Cario, Steve Hoffmann, Anke K. Bergmann

**Affiliations:** 1 Department of Human Genetics, Hannover Medical School (MHH), Hannover, Germany; 2 Hoffmann Group, Leibniz Institute on Aging - Fritz Lipmann Institute (FLI), Jena, Germany; 3 Department of Pediatrics, Berlin-Frankfurt-Münster ALL Study Group Germany (BFM-G), University Medical Center Schleswig-Holstein, Campus Kiel, Kiel, Germany; 4 Department of Pediatric Oncology and Hematology, Charité University Medical Center, Berlin, Germany; 5 Medical Faculty, Otto-von-Guericke-University Magdeburg, Magdeburg, Germany; 6 Department of Pediatric Hematology and Oncology, Hannover Medical School (MHH), Hannover, Germany

**Keywords:** Cancer genetics, Risk factors, Acute lymphocytic leukaemia

## To the Editor:

The dicentric chromosome dic(9;20)(p11∼13;q11) occurs in around 2% of pediatric ALL cases, almost exclusively of B-cell lineage [1–3]. Previous studies have demonstrated a lack of specific breakpoint clusters, indicating that the mechanism driving leukemogenesis in dic(9;20) may involve alterations in multiple genes. For example, highly recurrent alterations in several ALL-driving genes, including *CDKN2A*, *CDKN2B*, and *PAX5*, suggest that the loss of genetic material, rather than a specific genomic fusion, may be the underlying cause of leukemogenesis in dic(9;20) ALL [2–6]. However, the exact mechanism in which dic(9;20) drives ALL development has not been fully elucidated. Furthermore, previous studies have shown discordant findings regarding the outcomes of patients with dic(9;20) ALL in different treatment protocols [1, 3, 7, 8]. The aim of this study was to unravel the demographic, clinical, prognostic, and molecular characteristics of the dic(9;20)-positive ALL in a cohort of 57 pediatric B-ALL patients.

Detection of dic(9;20) by conventional cytogenetic and molecular methods was the prerequisite for inclusion in the study. All 57 dic(9;20) ALL cases, and 56 cases for which RNA samples were available, were subjected to genome-wide array CGH and targeted RNA-sequencing analysis. In addition, we performed whole transcriptome, whole genome, and whole genome bisulfite sequencing (WGBS) for a selected number of cases with rearrangements involving the *DNMT3B* gene. Informed written consent was obtained from all patients or their legal guardians before enrollment in the study (Supplementary Methods).

Overall, we included 22 male and 35 female children, with a median age at diagnosis of three years (range: 1–17 years). All patients achieved remission, 11 experienced relapses, while two patients in remission were lost to follow-up. Median white blood cell count at diagnosis was 32.6 × 10^9^/L (range: 1.3–248.3 × 10^9^/L). Risk group classification based on minimal residual disease (MRD) measurement was available for 51 patients, all of which were MRD-MR (*n* = 35; 68.6%), or MRD-SR (*n* = 16; 31.4%) (Table 1 and Supplementary Table S1).

**Table 1:** Summary of clinical characteristics of patients included in the study.

	Number of cases	%
Gender		
male	22	38.6
female	35	61.4
Age		
<1 year	0	0
1–5 years	50	87.7
6–9 years	3	5.3
≥10 years	4	7
WBC count (x10^9^/L)		
<10	16	28.1
10–50	20	35.1
50–100	12	21
≥100	9	15.8
CNS involvement		
negative	50	87.7
positive	4	7
no data	3	5.3
Prednisone response		
good	53	93
poor	2	3.5
no data	2	3.5
MRD after induction		
MRD-negative	17	29.8
MRD-positive	40	70.2
no data	0	0
MRD after consolidation		
MRD-negative	40	70.2
MRD-positive	9	15.8
no data	8	14
MRD-based risk group		
MRD-SR	16	28.1
MRD-MR	35	61.4
MRD-HR	0	0
no data	6	10.5
Risk group		
SR	15	26.3
MR	37	64.9
HR	5	8.8

*WBC* white blood cells, *CNS* central nervous system, *MRD* minimal residual disease, *SR* standard-risk, *MR* medium-risk, *HR* high-risk.

Our analysis of karyotype, array CGH copy number profiles, and chimeric fusion transcripts revealed the presence of deletions in the *CDKN2A* and *CDKN2B* genes in all 57 cases, as well as *PAX5* gene alterations in 56 cases (98%), which included 35 cases with *PAX5* gene deletions (63%; Supplementary Fig. S1A). Deletions of the *IKZF1* gene were found in 20 patients (35%), seven of which (35%) experienced relapse. Together with the complete absence of *ERG* deletions, the concomitant *IKZF1* and *CDKN2A*, *CDKN2B*, *PAX5*, or pseudoautosomal region 1 (PAR1) deletions suggest a strong association of dic(9;20) with the unfavorable prognostic marker IKZF1^plus^ [9]. Additional copies of chromosome 21 were the most common (*n* = 16; 28%), while other recurrent aneuploidies included additional copies of chromosomes 8 (*n* = 4), X (*n* = 4), 18 (*n* = 3) and 10 (*n* = 2), in line with findings in previous studies describing dic(9;20) ALL [1, 2, 4, 8]. In addition to dic(9;20), *CRLF2::P2RY8* fusions were found in five cases. Chimeric fusion transcripts involving *PAX5* and *C20orf112* (*n* = 10) were the most frequent in the cohort, followed by *ZCCHC7* and *DNMT3B* (*n* = 3). In addition to genomic rearrangements of *DNMT3B* with *ZCCHC7*, our RNA-seq and array CGH analysis revealed recurrent genomic rearrangements between *DNMT3B* and *PAX5* genes (*n* = 3), the latter being located adjacent to the *ZCCHC7* gene (Supplementary Fig. S1B). All three patients with genomic rearrangements between the *DNMT3B* and *PAX5* genes, as well as one patient with a rearrangement involving the *ZCCHC7* gene experienced relapse (Supplementary Fig. S2). Overall, these data illustrate the heterogeneous spectrum of genetic alterations in the cases with dic(9;20)-positive ALL.

**Fig. 1:**
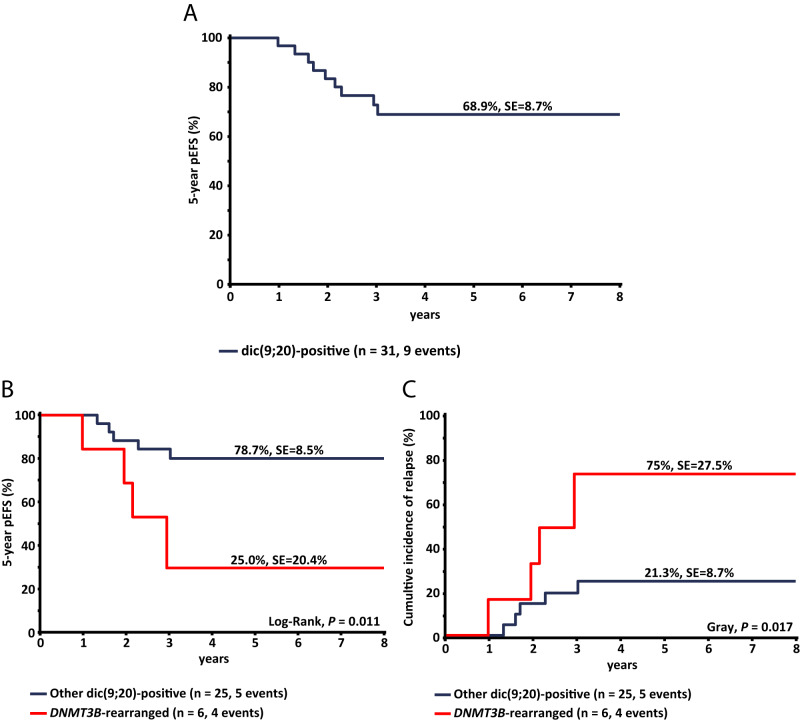
Outcome of the dic(9;20)-positive ALL cases. Predicted 5-year event-free survival (pEFS) in the total cohort of dic(9;20)-positive ALL cases (**A**), and 5-year pEFS in dic(9;20)-positive ALL cases with and without *DNMT3B* rearrangements (**B**). Cases with *DNMT3B* rearrangements have significantly worse 5-year pEFS, compared to the rest of the cohort (25% VS 79%, *P* = 0.011). Cumulative incidence of relapse (CIR) in dic(9;20)-positive ALL cases with and without *DNMT3B* rearrangements (**C**), indicating significantly higher CIR in cases with *DNMT3B* rearrangements compared to the rest of the cohort (75% VS 21.3%, *P* = 0.017). In order to restrict survival analyses to cases with sufficient follow-up time, only cases involved in the AIEOP-BFM 2000 and 2009 treatment studies were included. Curves were compared using the Log-rank and Gray tests.

Survival analyses were restricted to patients enrolled in the AIEOP-BFM ALL 2000 and 2009 studies, because these patients had sufficient follow-up time (*n* = 31; median follow-up 6.1 years). All patients achieved complete remission, one was lost to follow-up, and nine experienced relapse, resulting in an estimated probability of 5-year event-free survival (pEFS) of 69% (SE = 9%; Fig. 1A and Supplementary Table S1). Among the patients who relapsed, the majority were stratified in a medium-risk treatment arm (*n* = 6; 66%), and the majority were MRD-negative at the end of consolidation (*n* = 5; 56%; Supplementary Tables S1 and S2). Notably, CNS involvement at relapse was observed in six cases (66%), all of whom were CNS-negative at initial diagnosis. In the entire group of six cases with rearrangements in the *DNMT3B* and *PAX5* or *ZCCHC7* genes, four experienced relapses, resulting in a 5-year pEFS of 25% (SE = 20%), compared to 79% (SE = 9%) for the remaining cohort (*P* = 0.011; Fig. 1B and Supplementary Table S1). The CIR at five years was 75% (SE = 28%) and 21% (SE = 9%) for the patients with rearrangements in the *DNMT3B* gene and the remainder of the cohort, respectively (*P* = 0.017; Fig. 1C and Supplementary Table S1).

All six patients were treated according to the medium risk arm of the respective AIEOP-BFM ALL 2000 or 2009 protocols (*n* = 1 and *n* = 5, respectively), and the majority of patients were MRD-negative (*n* = 4; 67%), after consolidation (Supplementary Tables S1 and S2). In line with our observation for other dic(9;20)-positive ALL cases, the majority of relapses presented with CNS involvement (75%; Supplementary Table S3).

The rearrangement of *DNMT3B* and *ZCCHC7* (*n* = 3) resulted in an in-frame chimeric fusion transcript, containing the 5’ end of the open reading frame of *DNMT3B* and the 3’ end of *ZCCHC7* (Supplementary Figs. S3A and S3B and Supplementary Table S4). In contrast, due to the opposing orientation of the *DNMT3B* and *PAX5* genes, the resulting chimeric transcript contained the 5’ end of the open reading frame of *DNMT3B* or *PAX5* and a run-through transcript of the antisense strand of the partner gene (Supplementary Figs. S3A and S3C and Supplementary Table S4). DNMT3B is a de novo DNA methyltransferase, and deletions of this gene have been previously reported in various solid malignancies and AML [10]. Furthermore, it has been shown that the loss of the *DNMT3B* gene causes demethylation at specific CpG loci, as well as global hypomethylation in mouse embryonic stem cells [11, 12]. Since the methyltransferase domain of the *DNMT3B* gene was lost in five ALL with the *DNMT3B* rearrangements (Supplementary Fig. S3A and B), we hypothesized that the loss of methyltransferase activity might lead to specific as well as global methylation changes in the ALL with *DNMT3B* rearrangements. Therefore, we performed WGBS of ALL samples of all six patients with *DNMT3B* rearrangements, and, in addition, included four age-matched *ETV6::RUNX1*-positive ALL patients. However, our analysis did not show substantial changes in the global methylation levels (Supplementary Fig. S4A–C).

Interestingly, in all four cases that experienced relapse, breakpoints occurred in introns 6 and 7 of the *DNMT3B* gene. In contrast, in the remaining two non-relapsed cases, breakpoints occurred in introns 1 and 22, representing the first and last introns of the *DNMT3B* gene (Supplementary Fig. S3A). Clustering of the breakpoints in the *DNMT3B* gene in cases that relapsed, as well as the presence of the non-canonical fusion transcripts, suggest that leukemogenesis in these cases might be driven by a perturbation of the genomic regulatory mechanisms located in the vicinity of the breakpoints, rather than an aberrant function of the chimeric protein. Indeed, our examination of the genomic loci, corresponding to the breakpoint cluster in the introns 6 and 7 of the *DNMT3B* gene, revealed the presence of a weak B-cell-specific enhancer in intron 7 of the *DNMT3B* gene [13, 14], which overlapped with the DNase hypersensitivity region identified in the Blueprint project (Supplementary Methods and Supplementary Fig. S5). This data suggests that the loss or a hijack of this enhancer may be responsible for the perturbation of biological and molecular mechanisms driving leukemogenesis in the dic(9;20)-positive ALL cases with rearrangements in the introns 6 and 7 of the *DNMT3B* gene. In order to identify the genes whose regulation might be perturbed, we performed differential gene expression analysis between dic(9;20)-positive ALL cases with *DNMT3B* rearrangements involving introns 6 and 7 and those with breakpoints in other introns. Our analysis identified 143 significantly upregulated and 174 downregulated genes (*P* ≤ 0.1) in the dic(9;20)-positive ALL cases with *DNMT3B* rearrangements involving introns 6 and 7 (Supplementary Fig. S6 and Supplementary Table S5). The most significantly differentially expressed protein coding genes were *DAPK1* (located on chromosome 9 and upregulated) and *EPAS1* (downregulated), while the largest fold changes were observed for the genes *TCL1B* (upregulated) and *CNTN2* (downregulated). Interestingly, two of the upregulated genes in our analysis and known oncogenes, *TCL1B* and *HCK*, as well as two downregulated genes, *EPAS1* and *PTPN3*, are involved in hematopoiesis and lymphocyte activation [15] (Supplementary Fig. S6 and Supplementary Table S5), suggesting that the mechanisms in which dic(9;20)-positive ALL cases with rearrangements in the introns 6 and 7 of the *DNMT3B* gene drive leukemogenesis, may entail dysregulation of the genes and pathways involved in hematopoiesis, lineage commitment and activation of mature lymphocytes.

Our data shows that ALL with dic(9;20) alterations confer poor prognosis compared to BCP-ALL patients treated according to AIEOP-BFM 2000 and 2009 protocols. This was particularly the case with the dic(9;20)-positive ALL with *DNMT3B* rearrangements, which have poor outcome compared to the dic(9;20)-positive ALL without these alterations. Thus, once confirmed in an independent cohort, dic(9;20)-positive cases with rearrangements involving *DNMT3B* and *PAX5* or *ZCCHC7* genes should be considered high-risk for relapse and treated accordingly.

### Supplementary information

Supplementary Data

Supplementary Tables

## Data Availability

High-throughput sequencing and Array CGH data are available in the European Genome-Phenome Archive under accession number: EGAS00001007383.
