# Supplementary material for: Recurrent DNMT3B rearrangements are associated with unfavorable outcome in dicentric (9;20)-positive pediatric BCP-ALL
Source: Leukemia. 2023 Oct 16;37(12):2522–5. doi: 10.1038/s41375-023-02058-w (PMC10681884; doi:10.1038/s41375-023-02058-w)
Supplement: Supplementary file 1 — Supplementary Data [file 41375_2023_2058_MOESM1_ESM.docx]

**Recurrent *DNMT3B* rearrangements are associated with unfavorable outcome in dicentric (9;20)-positive pediatric BCP-ALL**

Željko Antić^1,2^, Alena van Bömmel^2^, Konstantin Riege^2^, Jana Lentes^1^, Charlotte Schröder^1^, Julia Alten^3^, Cornelia Eckert^4^, Lara Fuhrmann^1^, Doris Steinemann^1^, Lennart Lenk^3^, Denis M. Schewe^5^, Martin Zimmermann^6^, Martin Schrappe^3^, Brigitte Schlegelberger^1^, Gunnar Cario^3^, Steve Hoffmann^2^ and Anke K. Bergmann^1^

1. Department of Human Genetics, Hannover Medical School (MHH), Hannover, Germany
2. Leibniz Institute on Aging - Fritz Lipmann Institute (FLI), Jena, Germany
3. Department of Pediatrics, Berlin-Frankfurt-Münster ALL Study Group Germany (BFM-G), University Medical Center Schleswig-Holstein, Campus Kiel, Kiel, Germany
4. Department of Pediatric Oncology and Hematology, Charité University Medical Center, Berlin, Germany
5. Medical Faculty, Otto-von-Guericke-University Magdeburg, Magdeburg, Germany
6. Department of Pediatric Hematology and Oncology, Hannover Medical School (MHH), Hannover, Germany

**SUPPLEMENTARY METHODS**

***Cohort description***

The present study investigates a cohort of 57 children with dic(9;20)-positive B-ALL (22 male and 35 female), enrolled in the AIEOP-BFM 2000, 2009, or 2017 trials, who underwent cytogenetic diagnostic testing in our center (Table 1 and Supplementary Table S1). Detection of dic(9;20) alteration was performed using karyotyping (n=38; 71%) and Array CGH (n=57; 100%). Median age at diagnosis was 3 years (range: 1-17 years), and median follow-up time was 6.1 years. Informed written consent was obtained from all patients or their legal guardians before enrollment in the study. The study and use of ALL samples was approved by the institutional review board of the Medical Faculty of the Christian-Albrechts-University Kiel (BFM-ALL 2000: B257/01; AIEOP-BFM ALL 2009: A177/09; AIEOP-BFM ALL 2017: A105/18).

***DNA and RNA isolation***

DNA was isolated from mononuclear cells obtained from bone marrow aspirate or peripheral blood at initial diagnosis, using QIAamp DNA Mini Kit (Qiagen, Hilden, Germany). RNA was isolated from bone marrow aspirate, obtained at the initial diagnosis, using the Chemagic 360 instrument (PerkinElmer, Waltham, MA, USA). The quality assessment of the isolated RNA was performed using Bioanalyzer RNA 6000 Nano kit (Agilent Technologies, Waldbronn, Germany). The study and use of ALL samples was approved by the institutional review board of the Medical Faculty of the Christian-Albrechts-University Kiel (BFM-ALL 2000: B257/01; AIEOP-BFM ALL 2009: A177/09; AIEOP-BFM ALL 2017: A105/18).

***Karyotyping and fluorescence R-banding***

Karyotyping and fluorescence R-banding were performed as previously described [[1,2]](https://paperpile.com/c/NQzlLj/pMQ0t+KPoqI). Chromosome analysis was performed using Axioplan 2 imaging and Axio Imager.Z2 microscopes (Zeiss, Jena, Germany).

***Array CGH***

Array CGH analysis was performed by hybridizing 500ng of patient DNA using a 400K SurePrint G3 Custom CGH Human Genome Microarray (e-Array design 84704, Agilent Technologies, Waldbronn, Germany), as previously described [[1]](https://paperpile.com/c/NQzlLj/pMQ0t).

***RNA sequencing***

Targeted RNA sequencing was done with 100ng of input RNA, using TruSight RNA Pan-Cancer Panel (Illumina, San Diego, CA, USA), according to the manufacturer’s instructions. Libraries were sequenced on a MiSeq sequencer using a V3 reagent kit. The average number of read pairs per sample was 3 565 301 (median: 3 687 288; range: 992 565 - 5 240 627; Supplementary Table S1).

Whole transcriptome sequencing was performed using the TruSeq Stranded Total RNA Library Prep Gold kit (Illumina, San Diego, CA, USA) and 200ng of input RNA, according to the manufacturer’s instructions and as previously described [[2]](https://paperpile.com/c/NQzlLj/KPoqI). Sequencing was done on an Illumina NovaSeq sequencer using S4 Flow Cell. The average number of read pairs per sample was 257 936 317 million (median: 245 905 730; range: 233 718 806 - 333 697 570; Supplementary Table S6).

We used Trimmomatic v0.39 [[3]](https://paperpile.com/c/NQzlLj/OVomz) (5nt sliding window approach, mean quality cutoff 20) for read quality trimming according to inspections made from FastQC v0.11.9 reports. Illumina TruSeq universal adapter and anchored mono- and di-nucleotide content were clipped using Cutadapt v2.10 [[4]](https://paperpile.com/c/NQzlLj/DvmkH). Subsequently, ribosomal RNA (rRNA) transcripts were artificially depleted by read alignment against rRNA databases as performed by SortMeRNA v2.1 [[5]](https://paperpile.com/c/NQzlLj/db9Nc).

Gene fusion detection was performed on alignments derived from the mapping tool STAR v2.72b [[6]](https://paperpile.com/c/NQzlLj/WHvar) using GRCh38 CTAT genome lib Apr062020 as reference and parameter settings according to version-specific authors' recommendations of Arriba v1.2 [[7]](https://paperpile.com/c/NQzlLj/5KZOT) or STAR-Fusion v1.9.1 [[8]](https://paperpile.com/c/NQzlLj/GmLr7). FusionInspector-validated chimeric transcripts were complemented by predictions made from FusionCatcher v1.20 [[9]](https://paperpile.com/c/NQzlLj/1OCKb) (human database v98), given unprocessed sequencing data, and subsequently filtered for gene fusions supported by at least ten split junctions and spanning reads.

The pre-preprocessed sequencing data were aligned to the reference genome GRCh38, retrieved with its gene annotation from Ensembl v102 [[10]](https://paperpile.com/c/NQzlLj/tspXK) to examine gene expression. For this purpose, we used the mapping software segemehl v0.3.4 [[11,12]](https://paperpile.com/c/NQzlLj/rlK9U+bGCJI) with adjusted accuracy (95%) and enabled split-read mode. Mappings were filtered by Samtools v1.14 [[13]](https://paperpile.com/c/NQzlLj/Xkw02) for uniqueness and properly aligned mate pairs. Quantification was performed on exon level (10 nt minimum overlap) using featureCounts v2.0.1 [[14]](https://paperpile.com/c/NQzlLj/xcO9Z), parameterized to match the samples library strand specificity, inferred using RSeQC v4.0.0 [[15]](https://paperpile.com/c/NQzlLj/E7gEC). Differential gene expression analysis for samples with *DNMT3B* rearrangements was done using DESeq2 v1.34.0 [[16]](https://paperpile.com/c/NQzlLj/qpln9). The null hypothesis of the equality of gene expression was rejected whenever an adjusted *P*-value of lower than 0.1 was found. The heatmap with variance stabilized gene counts calculated using DESeq2 function vst was made using the ComplexHeatmap v2.10.0 [[17]](https://paperpile.com/c/NQzlLj/Pk3a2) library in R.

***Whole genome sequencing***

Whole genome sequencing was performed using Lotus DNA Library Prep Kit (Illumina, San Diego, CA, USA) with 250ng of input DNA, according to the manufacturer’s instructions. Sequencing was done on an Illumina NovaSeq sequencer using S4 Flow Cell. We achieved an average sequencing depth of 42.4x (median: 43.8x; range: 31.3-49.4x; Supplementary Table S6).

Analogous to the RNA sequencing data, WGS samples were subjected to quality and adapter assessment as described above and subsequently aligned to the reference genome GRCh38 using BWA-mem2 v2.2.1 [[18]](https://paperpile.com/c/NQzlLj/g8hLg), parameterized according to a minimum alignment output score 40 and enabled soft-clipping. Paired primary alignments were selected via Samtools v1.14 [[13]](https://paperpile.com/c/NQzlLj/Xkw02). Duplicated reads were excluded using SAMBLASTER v0.1.26 [[19]](https://paperpile.com/c/NQzlLj/jCxxB) while extracting discordant mate pairs and split (i.e., supplementary) alignments for downstream breakpoint detection to inspect the origin of observed *DNMT3B* fusion transcripts. Structural variant calling was performed by a combinatorial method that unifies significant inter-chromosomal break ends, detected at *DNMT3B* locus, from five tools: GRIDSS2 v2.12.0 [[20]](https://paperpile.com/c/NQzlLj/rYTvJ), SvABA v1.1.0 [[21]](https://paperpile.com/c/NQzlLj/jj2yv), LUMPY v0.3.1 [[22]](https://paperpile.com/c/NQzlLj/42aVg), Wham v1.8.0 [[23]](https://paperpile.com/c/NQzlLj/3lMs7) and DELLY v0.8.7 [[24]](https://paperpile.com/c/NQzlLj/zSRyY) using the ENCODE [[25]](https://paperpile.com/c/NQzlLj/9ksNR) blacklist ENCFF356LFX [[26,27]](https://paperpile.com/c/NQzlLj/6yljY+rGtUy).

***Whole genome bisulfite sequencing***

The DNA methylome was assessed using Ultralow Methyl-Seq with TrueMethyl oxBS Module (Tecan, Männedorf, Switzerland), according to the manufacturer’s instructions. In total, 300ng of fragmented DNA (200-300 bp fragment size) was used as an input for subsequent steps. Sequencing was performed using the NovaSeq 6000 platform. We achieved an average sequencing depth of 49.6x (median: 49x; range 47.7-52.1x; Supplementary Table S6).

WGBS datasets were pre-processed as described above and aligned to GRCh38 genome reference using segemehl v0.3.4 in methylC-seq mode, and mapping accuracy was adjusted to 95%. Uniquely and properly aligned mate pairs were extracted via Samtools v1.14 and filtered for duplicated reads with Picard MarkDuplicates v2.23.8. Further, overlapping mate pair sequences were cut from the second mate using BamUtil clipOverlap v1.0.14 [[28]](https://paperpile.com/c/NQzlLj/cZbP5). To infer methylation rates for all cytosines within a CpG context with a read coverage of at least 10, the data-adaptive variant caller haarz v0.3.0 [[29]](https://paperpile.com/c/NQzlLj/UkmIl) was used. Finally, BEDTools unionbedg v2.30.0 [[30]](https://paperpile.com/c/NQzlLj/GBZqQ) was used to compile matrices of methylation rates. Principal component analysis (PCA) was calculated on all cytosines covered in all samples (n = 4,940,688) using prcomp function in R stats v4.1.3 package. The distribution in the methylation intervals (<0.25, 0.25-0.5, 0.5-0.75, 0.75-0.95, ≥0.95) was calculated on all cytosines covered in at least one sample (n = 27,637,217). First, the mean methylation value per group (*DNMT3B*-positive with breakpoints in intron 6 or 7, *DNMT3B*-positive with breakpoints in other introns, and *ETV6::RUNX1*-positive) in each cytosine was calculated and assigned to one of the five methylation intervals. The relative frequencies were then plotted using the R library ggplot2 v 3.4.2.

***Breakpoint-spanning PCR and Sanger sequencing***

cDNA synthesis was performed with RevertAid First Strand cDNA Synthesis Kit (ThermoFisher Scientific, Waltham, MA, USA), using 150ng of input RNA. PCR reactions were done with 10ng of input DNA, or cDNA, using AmpliTaq Gold kit (ThermoFisher Scientific, Waltham, MA, USA), and purified using ExoSAP-IT PCR Product Cleanup Reagent (ThermoFisher Scientific, Waltham, MA, USA), or NucleoSpin Gel and PCR Clean‑up Mini kit (Macherey-Nagel, Duren, Germany). Primers were designed using primer3 software. Sanger sequencing, with appropriate forward or reverse primer, was performed by Microsynth Seqlab (Göttingen, Germany). List of primers used is available in the Supplementary Table S4.

***Statistical testing***

Categorical variables were compared using Fisher exact test. Event-free survival (EFS) was estimated according to Kaplan-Meier, and the curves were compared by log-rank test. The cumulative incidence of relapse was calculated according to Kalbfleisch and Prentice [[31]](https://paperpile.com/c/NQzlLj/gRJLK) and compared with the Gray test. Survival analyses were restricted to patients with sufficient follow-up time (patients involved in the AIEOP-BFM ALL 2000 and 2009 treatment studies).

**SUPPLEMENTARY FIGURES**

**
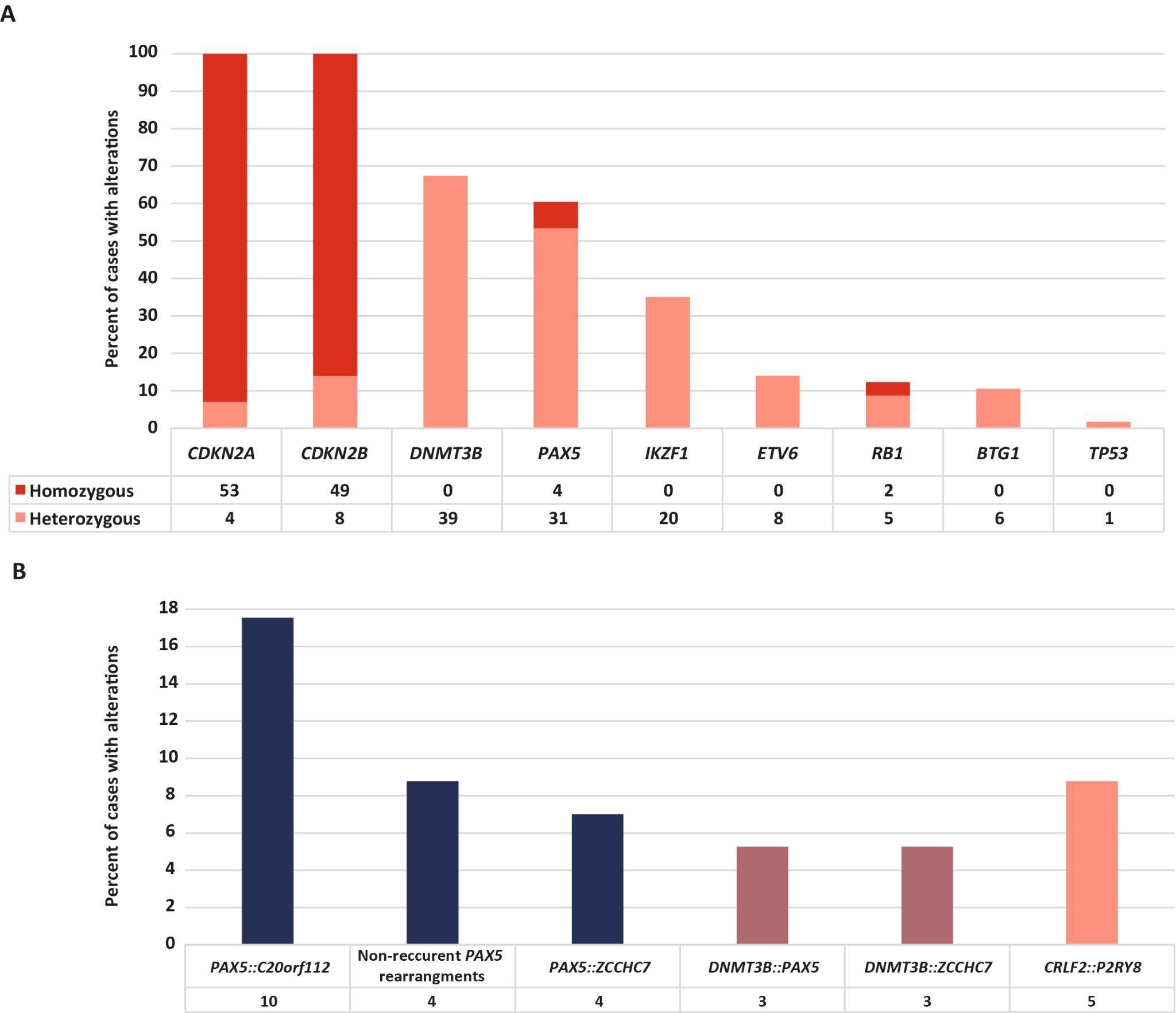
**

**Supplementary Figure S1 - Frequency of the recurrent genomic alterations in the dic(9;20)-positive ALL cases.**

Bar graphs depicting the frequency of the recurrent copy number alterations **(A)** and gene fusions **(B)** in the cohort of dic(9;20)-positive ALL cases. Bars are sorted based on the frequency and the type of genomic alterations. Tables below each bar graph show absolute numbers of cases with respective alterations. Cases with rearrangements in *PAX5* and *DNMT3B* genes are excluded from the plot depicting copy number alterations, as none of the cases with rearrangements in these genes had deletion of the second allele.


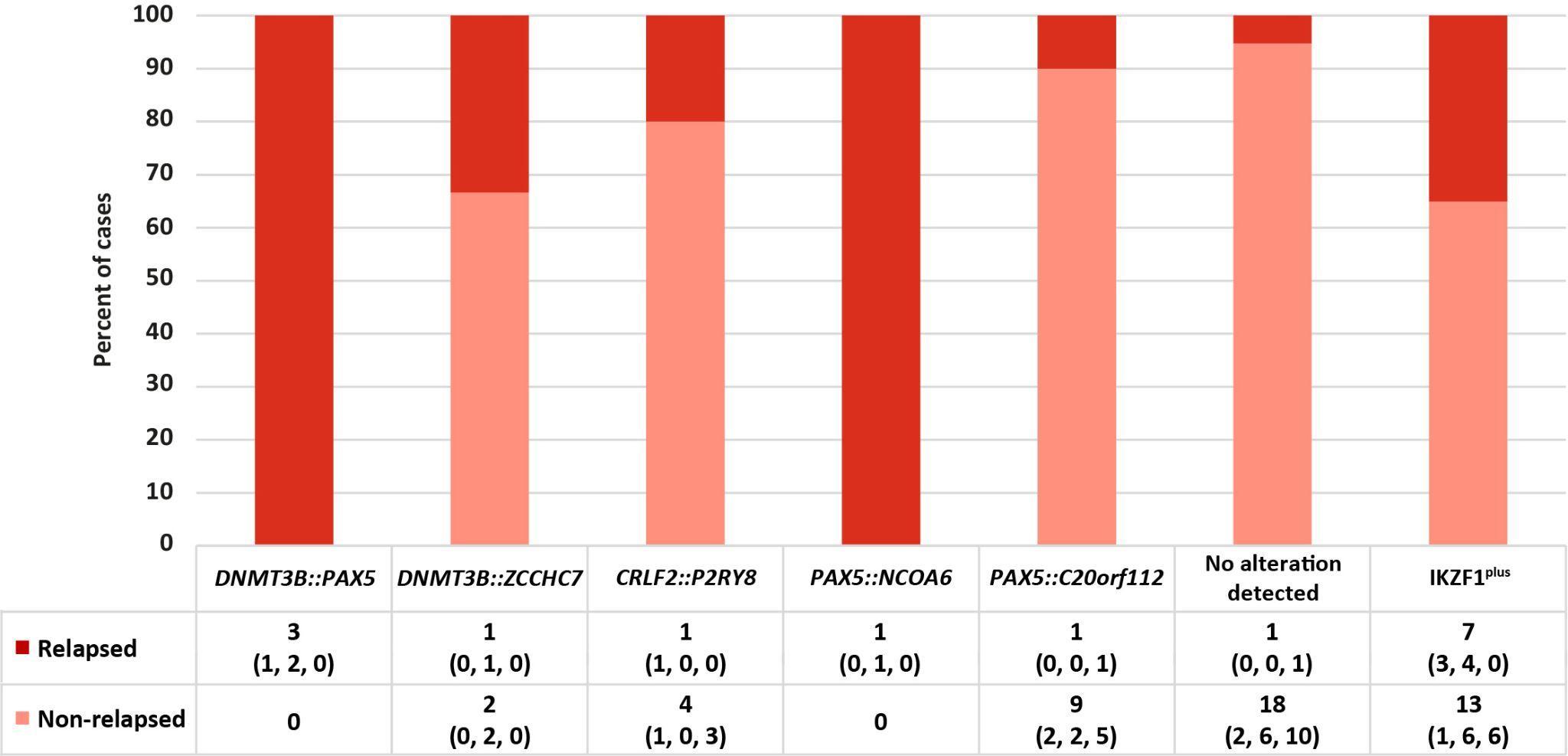


**Supplementary Figure S2 – Frequency of relapse in the dic(9;20)-positive ALL cases.**

Bar graph showing the distribution of relapsed and non-relapsed cases with genomic alterations detected in the study. Only genomic alterations with at least one relapse event are shown. The table below the bar graph shows absolute numbers. Distribution of cases in the AIEOP-BFM ALL 2000, 2009 and 2017 treatment studies are shown in the brackets, respectively.

**
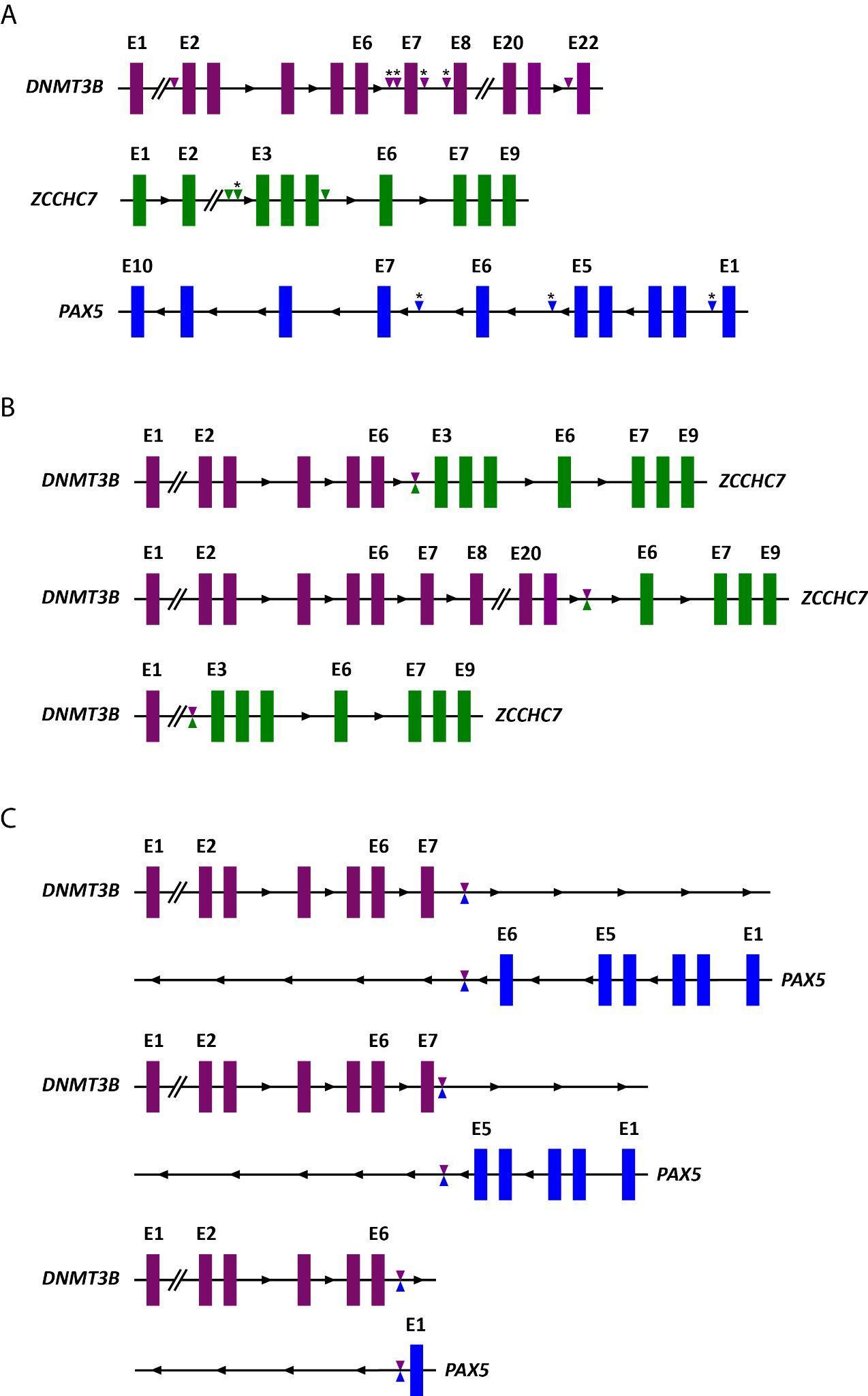
**

**Supplementary Figure S3 - Genomic breakpoints in the *DNMT3B* and partner genes *ZCCHC7* and *PAX5*.**

Schematic representation of the genomic breakpoints detected in the *DNMT3B*, *ZCCHC7* and *PAX5* genes **(A)**. Positions of the breakpoints are marked with a triangle, while cases that relapsed are marked with an asterisk above the triangle. Methyltransferase domain of the *DNMT3B* gene is encoded by exons 16-19. **(B)** Schematic representation of the chimeric fusion transcripts in cases with the rearrangements involving *ZCCHC7* gene. **(C)** Schematic representation of the chimeric fusion transcripts in cases with rearrangements involving *PAX5* gene.

**
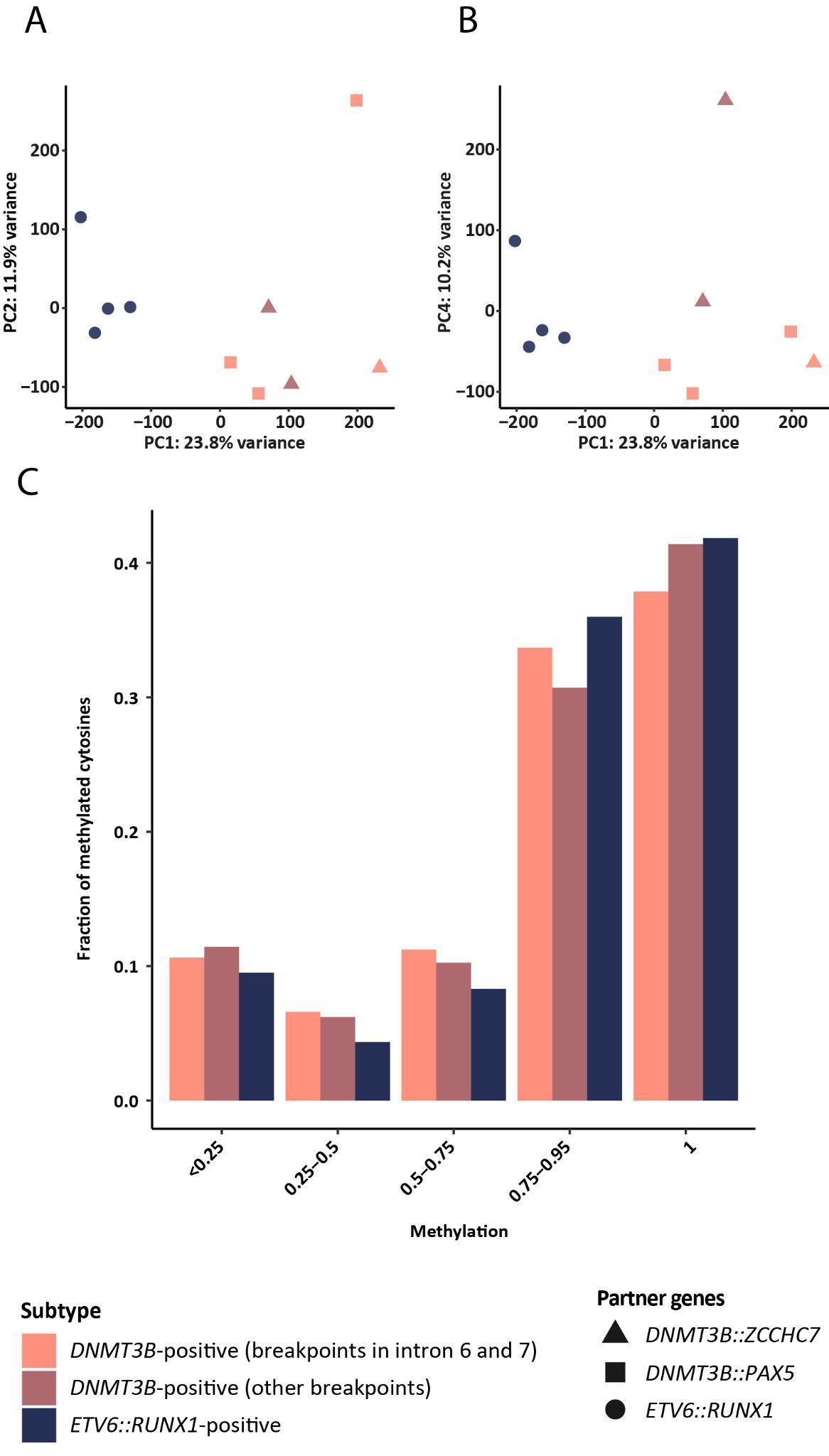
**

**Supplementary Figure S4 - Loss of the methyltransferase domain of *DNMT3B* gene does not lead to extensive changes in the global methylation patterns.**

Principal component analysis of CpG methylation, showing three most variable principal components, PC1 and PC2 **(A)** and PC1 and PC4 **(B)**, in *DNMT3B*-rearranged ALL with breakpoints in intron 6 and 7 of *DNMT3B* gene, *DNMT3B*-rearranged ALL with breakpoints in other introns, and *ETV6::RUNX1*-positive ALL. Samples with *DNMT3B* rearrangements formed a separate cluster from *ETV6::RUNX1*-positive samples, however, separation between two groups of samples with *DNMT3B* rearrangements was less distinctive. Relative methylation in three groups of samples **(C)**, indicating similar global methylation patterns between *DNMT3B*-rearranged and *ETV6::RUNX1*-positive ALL.


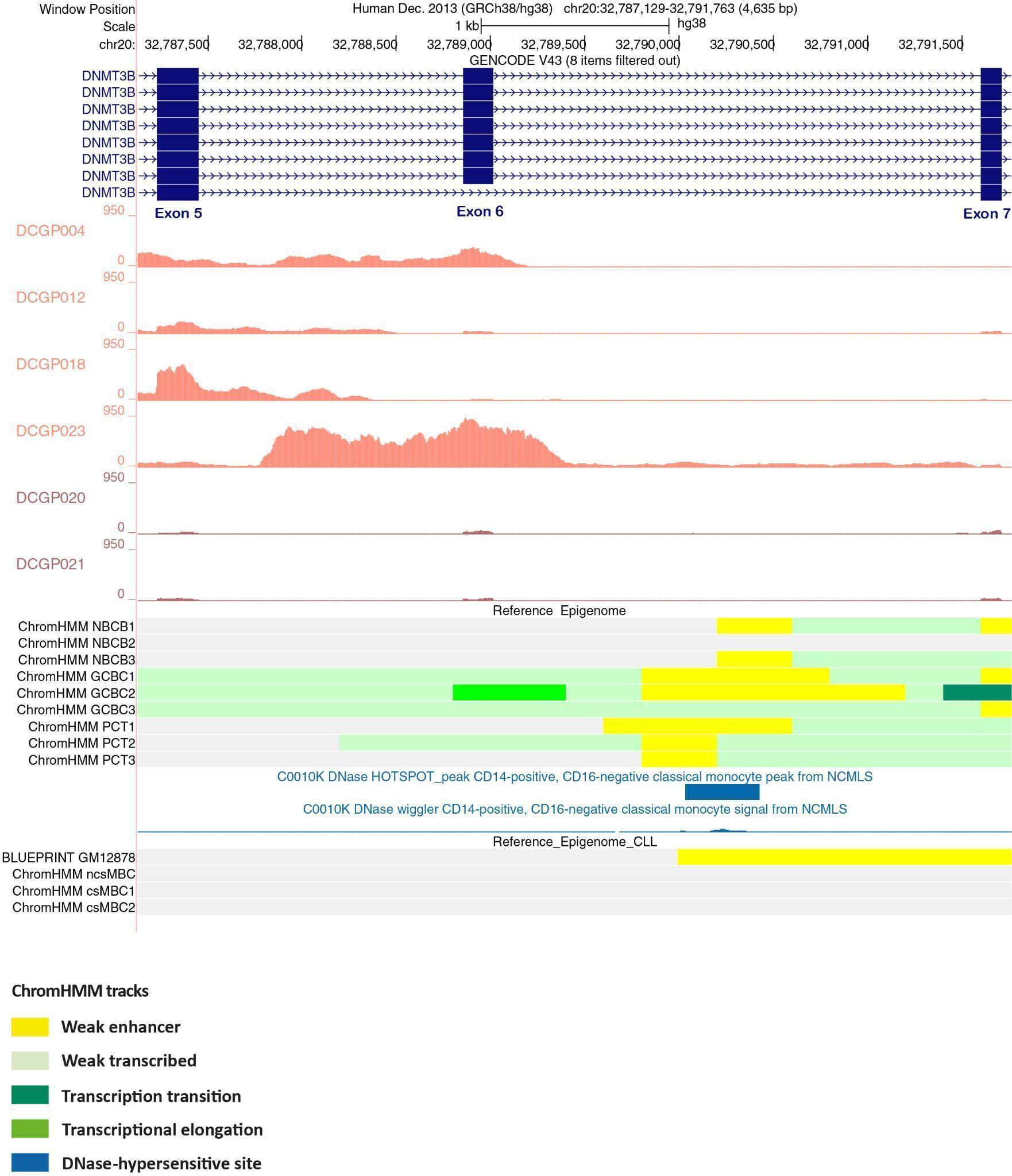


**Supplementary Figure S5 – Inspection of the breakpoints in the introns 6 and 7 of the *DNMT3B* gene reveals presence of the weak enhancer.**

Top panel shows schematic representation of the *DNMT3B* gene as depicted in the USCS browser. Shown below are whole transcriptome coverage tracks from six patients with dic(9;20) ALL and *DNMT3B* gene rearrangements. Patients with breakpoints in introns 6 and 7 of the *DNMT3B* gene are shown in light orange, while patients with other breakpoints are depicted in dark orange. ChromHMM tracks reveal the presence of the weak enhancer in B-cells (depicted in yellow color). The bottom panel (in blue) depicts the presence of the DNAse hypersensitivity site identified in monocytes in the Blueprint project (August 2016 data release).

Abbreviations: GCBC - Germinal center B-cell; NBCB - Naïve B-cell; PCT - Plasma B-cells.

**
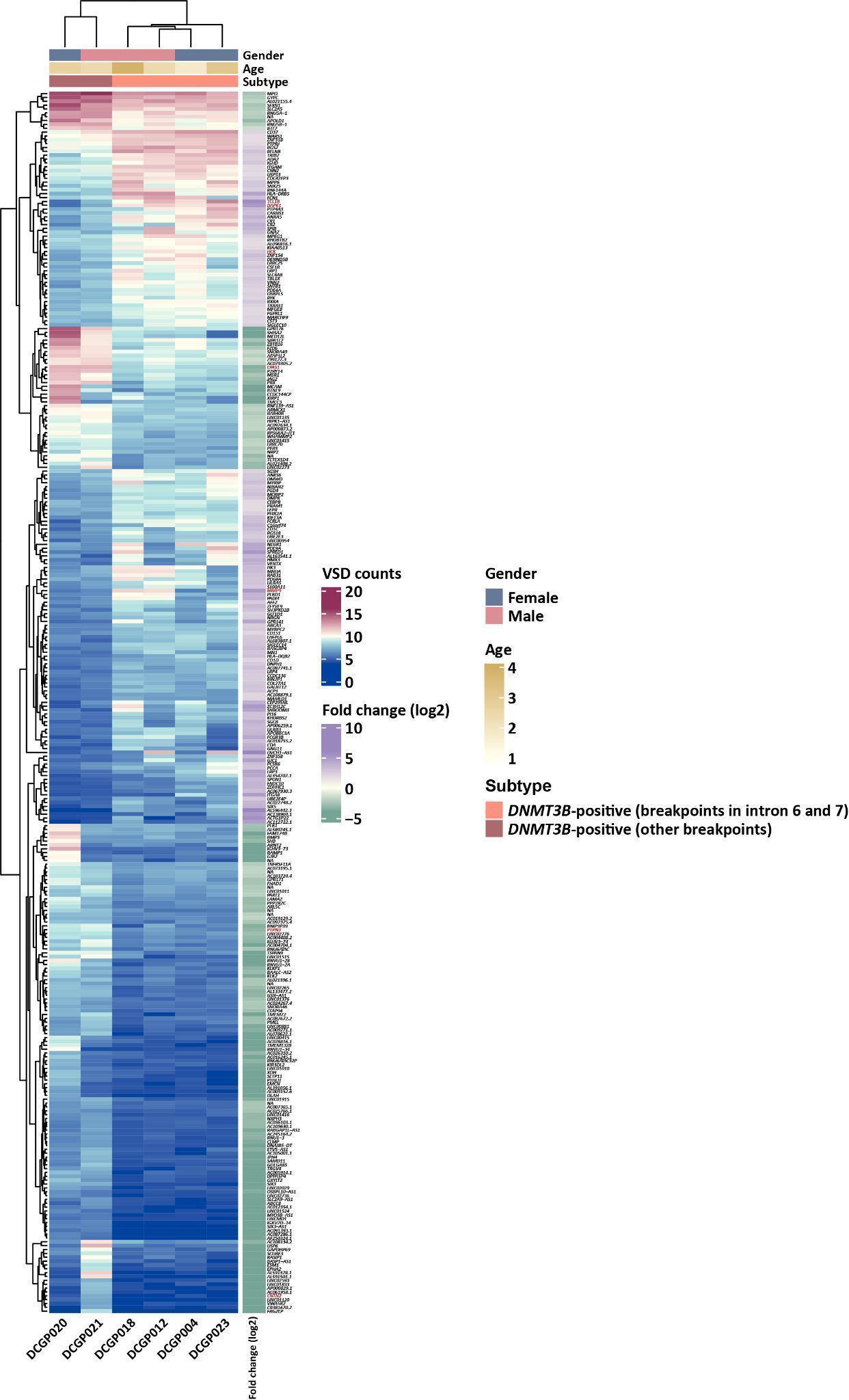
**

**Supplementary Figure S6 – Heatmap of differentially expressed genes between two groups of dic(9;20) ALL with *DNMT3B* rearrangements.**

Heatmap depicts top upregulated and downregulated genes between dic(9;20) ALL with *DNMT3B* rearrangements and breakpoints in introns 6 and 7 of the *DNMT3B* gene, and those with breakpoints in other introns. Genes with the biggest fold change, or lowest *P*-value, discussed in the result section, are depicted in dark red.

**REFERENCES**

[1] [Schieck M, Lentes J, Thomay K, Hofmann W, Behrens YL, Hagedorn M, et al. Implementation of RNA sequencing and array CGH in the diagnostic workflow of the AIEOP-BFM ALL 2017 trial on acute lymphoblastic leukemia. Ann Hematol 2020;99:809–18.](http://paperpile.com/b/NQzlLj/pMQ0t)

[2] [Chouvarine P, Antić Ž, Lentes J, Schröder C, Alten J, Brüggemann M, et al. Transcriptional and Mutational Profiling of B-Other Acute Lymphoblastic Leukemia for Improved Diagnostics. Cancers 2021;13:5653.](http://paperpile.com/b/NQzlLj/KPoqI)

[3] [Bolger AM, Lohse M, Usadel B. Trimmomatic: a flexible trimmer for Illumina sequence data. Bioinformatics 2014;30:2114–20.](http://paperpile.com/b/NQzlLj/OVomz)

[4] [Martin M. Cutadapt removes adapter sequences from high-throughput sequencing reads. EMBnet J 2011;17:10.](http://paperpile.com/b/NQzlLj/DvmkH)

[5] [Kopylova E, Noé L, Touzet H. SortMeRNA: fast and accurate filtering of ribosomal RNAs in metatranscriptomic data. Bioinformatics 2012;28:3211–7.](http://paperpile.com/b/NQzlLj/db9Nc)

[6] [Dobin A, Davis CA, Schlesinger F, Drenkow J, Zaleski C, Jha S, et al. STAR: ultrafast universal RNA-seq aligner. Bioinformatics 2013;29:15–21.](http://paperpile.com/b/NQzlLj/WHvar)

[7] [Uhrig S, Ellermann J, Walther T, Burkhardt P, Fröhlich M, Hutter B, et al. Accurate and efficient detection of gene fusions from RNA sequencing data. Genome Res 2021;31:448–60.](http://paperpile.com/b/NQzlLj/5KZOT)

[8] [Haas BJ, Dobin A, Stransky N, Li B, Yang X, Tickle T, et al. STAR-fusion: Fast and accurate fusion transcript detection from RNA-Seq. bioRxiv 2017. https://doi.org/](http://paperpile.com/b/NQzlLj/GmLr7)[10.1101/120295](http://dx.doi.org/10.1101/120295)[.](http://paperpile.com/b/NQzlLj/GmLr7)

[9] [Nicorici D, Satalan M, Edgren H, Kangaspeska S, Murumagi A, Kallioniemi O, et al. FusionCatcher - a tool for finding somatic fusion genes in paired-end RNA-sequencing data. bioRxiv 2014. https://doi.org/](http://paperpile.com/b/NQzlLj/1OCKb)[10.1101/011650](http://dx.doi.org/10.1101/011650)[.](http://paperpile.com/b/NQzlLj/1OCKb)

[10] [Cunningham F, Allen JE, Allen J, Alvarez-Jarreta J, Amode MR, Armean IM, et al. Ensembl 2022. Nucleic Acids Res 2022;50:D988–95.](http://paperpile.com/b/NQzlLj/tspXK)

[11] [Hoffmann S, Otto C, Doose G, Tanzer A, Langenberger D, Christ S, et al. A multi-split mapping algorithm for circular RNA, splicing, trans-splicing and fusion detection. Genome Biol 2014;15:R34.](http://paperpile.com/b/NQzlLj/rlK9U)

[12] [Hoffmann S, Otto C, Kurtz S, Sharma CM, Khaitovich P, Vogel J, et al. Fast mapping of short sequences with mismatches, insertions and deletions using index structures. PLoS Comput Biol 2009;5:e1000502.](http://paperpile.com/b/NQzlLj/bGCJI)

[13] [Li H, Handsaker B, Wysoker A, Fennell T, Ruan J, Homer N, et al. The Sequence Alignment/Map format and SAMtools. Bioinformatics 2009;25:2078–9.](http://paperpile.com/b/NQzlLj/Xkw02)

[14] [Liao Y, Smyth GK, Shi W. featureCounts: an efficient general purpose program for assigning sequence reads to genomic features. Bioinformatics 2014;30:923–30.](http://paperpile.com/b/NQzlLj/xcO9Z)

[15] [Wang L, Wang S, Li W. RSeQC: quality control of RNA-seq experiments. Bioinformatics 2012;28:2184–5.](http://paperpile.com/b/NQzlLj/E7gEC)

[16] [Love MI, Huber W, Anders S. Moderated estimation of fold change and dispersion for RNA-seq data with DESeq2. Genome Biol 2014;15:550.](http://paperpile.com/b/NQzlLj/qpln9)

[17] [Gu Z, Eils R, Schlesner M. Complex heatmaps reveal patterns and correlations in multidimensional genomic data. Bioinformatics 2016;32:2847–9.](http://paperpile.com/b/NQzlLj/Pk3a2)

[18] [Vasimuddin M, Misra S, Li H, Aluru S. Efficient architecture-aware acceleration of BWA-MEM for multicore systems. 2019 IEEE International Parallel and Distributed Processing Symposium (IPDPS), IEEE; 2019, p. 314–24.](http://paperpile.com/b/NQzlLj/g8hLg)

[19] [Faust GG, Hall IM. SAMBLASTER: fast duplicate marking and structural variant read extraction. Bioinformatics 2014;30:2503–5.](http://paperpile.com/b/NQzlLj/jCxxB)

[20] [Cameron DL, Baber J, Shale C, Valle-Inclan JE, Besselink N, van Hoeck A, et al. GRIDSS2: comprehensive characterisation of somatic structural variation using single breakend variants and structural variant phasing. Genome Biol 2021;22:202.](http://paperpile.com/b/NQzlLj/rYTvJ)

[21] [Wala JA, Bandopadhayay P, Greenwald NF, O’Rourke R, Sharpe T, Stewart C, et al. SvABA: genome-wide detection of structural variants and indels by local assembly. Genome Res 2018;28:581–91.](http://paperpile.com/b/NQzlLj/jj2yv)

[22] [Layer RM, Chiang C, Quinlan AR, Hall IM. LUMPY: a probabilistic framework for structural variant discovery. Genome Biol 2014;15:R84.](http://paperpile.com/b/NQzlLj/42aVg)

[23] [Kronenberg ZN, Osborne EJ, Cone KR, Kennedy BJ, Domyan ET, Shapiro MD, et al. Wham: Identifying Structural Variants of Biological Consequence. PLoS Comput Biol 2015;11:e1004572.](http://paperpile.com/b/NQzlLj/3lMs7)

[24] [Rausch T, Zichner T, Schlattl A, Stütz AM, Benes V, Korbel JO. DELLY: structural variant discovery by integrated paired-end and split-read analysis. Bioinformatics 2012;28:i333–9.](http://paperpile.com/b/NQzlLj/zSRyY)

[25] [ENCODE Project Consortium. An integrated encyclopedia of DNA elements in the human genome. Nature 2012;489:57–74.](http://paperpile.com/b/NQzlLj/9ksNR)

[26] [Amemiya HM, Kundaje A, Boyle AP. The ENCODE Blacklist: Identification of Problematic Regions of the Genome. Sci Rep 2019;9:9354.](http://paperpile.com/b/NQzlLj/6yljY)

[27] [Wimberley CE, Heber S. PeakPass: Automating ChIP-Seq Blacklist Creation. J Comput Biol 2020;27:259–68.](http://paperpile.com/b/NQzlLj/rGtUy)

[28] [Jun G, Wing MK, Abecasis GR, Kang HM. An efficient and scalable analysis framework for variant extraction and refinement from population-scale DNA sequence data. Genome Res 2015;25:918–25.](http://paperpile.com/b/NQzlLj/cZbP5)

[29] [Hoffmann S, Stadler PF, Strimmer K. A simple data-adaptive probabilistic variant calling model. Algorithms Mol Biol 2015;10:10.](http://paperpile.com/b/NQzlLj/UkmIl)

[30] [Quinlan AR, Hall IM. BEDTools: a flexible suite of utilities for comparing genomic features. Bioinformatics 2010;26:841–2.](http://paperpile.com/b/NQzlLj/GBZqQ)

[31] [Prentice RL, Kalbfleisch JD, Peterson AV Jr, Flournoy N, Farewell VT, Breslow NE. The analysis of failure times in the presence of competing risks. Biometrics 1978;34:541–54.](http://paperpile.com/b/NQzlLj/gRJLK)
